# Stratum corneum dysfunction in dandruff

**DOI:** 10.1111/j.1468-2494.2012.00723.x

**Published:** 2012-08

**Authors:** G A Turner, M Hoptroff, C R Harding

**Affiliations:** Unilever Research & Development Port SunlightQuarry Road East, Bebington, Merseyside CH63 3JW, UK

**Keywords:** dandruff, fatty acids, *Malassezia*, scalp, stratum corneum

## Abstract

**Synopsis:**

Dandruff is characterized by a flaky, pruritic scalp and affects up to half the world’s population post-puberty. The aetiology of dandruff is multifactorial, influenced by *Malassezia*, sebum production and individual susceptibility. The commensal yeast *Malassezia* is a strong contributory factor to dandruff formation, but the presence of *Malassezia* on healthy scalps indicates that *Malassezia* alone is not a sufficient cause. A healthy stratum corneum (SC) forms a protective barrier to prevent water loss and maintain hydration of the scalp. It also protects against external insults such as microorganisms, including *Malassezia,* and toxic materials. Severe or chronic barrier damage can impair proper hydration, leading to atypical epidermal proliferation, keratinocyte differentiation and SC maturation, which may underlie some dandruff symptoms. The depleted and disorganized structural lipids of the dandruff SC are consistent with the weakened barrier indicated by elevated transepidermal water loss. Further evidence of a weakened barrier in dandruff includes subclinical inflammation and higher susceptibility to topical irritants. We are proposing that disruption of the SC of the scalp may facilitate dandruff generation, in part by affecting susceptibility to metabolites from *Malassezia*. Treatment of dandruff with cosmetic products to directly improve SC integrity while providing effective antifungal activity may thus be beneficial.

**Résumé:**

Les pellicules se caractérisent par un cuir chevelu prurigineux, squameux, et affectent jusqu’à la moitié de la population post-pubertaire du monde. L’étiologie des pellicules est multifactorielle, influencée par Malassezia, par la production de sébum, et par la susceptibilité individuelle. La levure commensale Malassezia est un facteur fortement contributif à la formation de pellicules, mais la présence de Malassezia aussi sur les cuirs chevelus sains indique que Malassezia seule n’est pas une cause suffisante. Un stratum corneum (SC) sain forme une barrière protectrice pour empêcher la perte d’eau et maintenir l’hydratation du cuir chevelu. Il protège également contre les agressions externes tels les micro-organismes, y compris Malassezia, ou des substances toxiques. Des dommages aigus ou chroniques au niveau de la barrière peuvent nuire à une bonne hydratation, conduisant à des effets atypiques de la prolifération épidermique, de la différenciation des kératinocytes, et de la maturation du SC, ce qui peut expliquer une partie des symptômes des pellicules. L’appauvrissement et la désorganisation des lipides structurels d’un stratum corneum sujet aux pellicules sont compatibles avec la notion d’une barrière affaiblie telle qu’indiquée par une perte d’eau transépidermique élevée. Une preuve supplémentaire d’une barrière affaiblie dans les cas des pellicules est fournie par un niveau d’inflammation infraclinique et une plus grande susceptibilité aux irritants topiques. Nous proposons que la perturbation du SC du cuir chevelu facilite la production de pellicules, en partie en augmentant la sensibilité aux métabolites de Malassezia. Le traitement des pellicules avec des produits cosmétiques pour améliorer directement l’intégrité du SC, tout en offrant une activité antifongique efficace peut donc être bénéfique.

## Introduction

**Figure 1:**
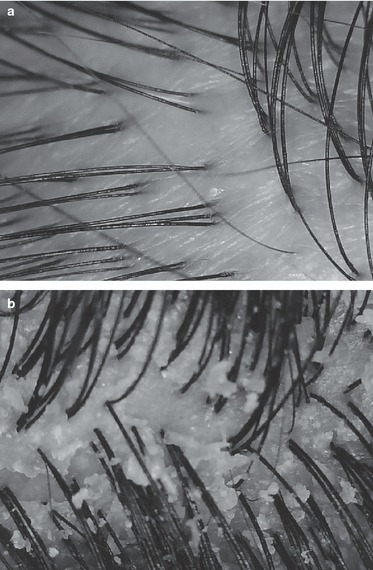
(a) Healthy Scalp (dandruff free). (b) Dandruff scalp showing characteristic flaking. Dandruff severity would be for this image is Grade c–d (see Table I).

Dandruff is a common complaint; as much as half the global population will suffer from the condition at some time [1]. Dandruff is generally characterized by the presence of flakes on the scalp and in the hair (Fig. 1), and is often accompanied by itch. The condition tends to develop post-puberty and is restricted to the scalp with the presence of terminal hair. The severity of dandruff can range from mild scale formation similar to dry skin to seborrhoeic dermatitis (SD) [2, 3]. Although often characterized as a more severe form of dandruff, there is currently no clear agreement on the symptomatic and aetiological links between these two conditions, leading to confusion between SD and dandruff [4–6].

The scientific consensus is that dandruff development is predicated by three major factors: *Malassezia* colonization, sebum production and individual predisposition. The interdependence of these factors underlies the timing and pattern of dandruff development. The sebaceous glands mature and produce greater amounts of sebum in both men and women at puberty [7]. The lipophilic yeast *Malassezia* utilizes sebum lipids as a nutrient source, and sebum production is hypothesized to be required to support growth of *Malassezia*. It has been widely theorized that this increase in sebum production and *Malassezia* proliferation triggers the development of dandruff.

Nevertheless, *Malassezia* is a commensal organism that is found on healthy scalps as well as dandruff scalps. The role of the yeast *Malassezia* in dandruff and SD has been proposed since it was first shown in 1874 that levels of *Malassezia* species are elevated in dandruff. For example, McGinley *et al.* found that *Malassezia* made up 46% of the microbial flora in normal subjects, 74% in patients with dandruff and 83% in cases of SD [8, 9]. Consistent with these observations, it is well established that the most effective antidandruff treatments are antifungal agents (e.g. zinc pyrithione, selenium sulphide and ketoconazole), improvement of the dandruff condition being correlated with removal of the yeast [10–12]. An investigation of the relative effectiveness of the known anti-*Malassezial* actives (selenium sulphide, zinc pyrithione and ketoconazole) did not show any significant differences in antimicrobial efficacy *in vivo* during product use. However, ketoconazole did show greater residual efficacy than the other two actives [11].

However, the commensal nature of *Malassezia* implies that there are other factors that make certain individuals more susceptible to the development of dandruff. The nature of this predisposition has been challenging to decipher, as many factors can affect scalp health, including environmental stresses such as climate and seasonality, microbial colonization and hormonal changes resulting in clinical and subclinical effects [13].

A critical, often-overlooked factor that may drive individual susceptibility to dandruff is the intrinsic quality of the scalp stratum corneum (SC). The SC is the major protective barrier against external insults (e.g. microbes, oxidative stressors, UV irradiation and toxic materials) and acts as the primary epidermal barrier to water loss, maintaining healthy hydration and integrity of the scalp.

In the dandruff scalp, the level of essential SC barrier lipids is reduced, the relative ratios altered [14] and structural organization compromised [15]. That these changes in lipid levels impact barrier quality is supported by the observation that transepidermal water loss (TEWL) in the dandruff scalp is greater than in the healthy scalp, which is further evidence of a perturbed barrier. In addition, the impaired barrier associated with the dandruff scalp exhibits an underlying propensity for hyperproliferation, altered corneocyte maturation processes and a subclinical inflammatory state [15, 16]. Understanding the biophysical, biochemical and ultrastructural changes in the properties of the SC that occur with dandruff may further elucidate the mechanisms of dandruff initiation or susceptibility.

In this review, we will discuss the recent evidence for the impact of individual predisposition, *Malassezia* colonization and sebum production on the development of dandruff. In particular, we will focus on the importance of SC barrier integrity in the aetiology of dandruff.

## Microorganisms and dandruff

### The scalp as a microbial habitat

Each region of the skin has a distinct ecological niche. The scalp represents a unique environment, with thick terminal hair, large numbers of sweat and sebaceous glands, and high relative humidity creating favourable conditions for microbial colonization. Maturation of the SC generates a steady supply of amino acids, sweat glands secrete minerals and sebaceous glands provide sebum, which act together to provide a nutrient-rich environment [17].

### *Malassezia* as a microbial contributor to dandruff

Although the precise link between *Malassezia* and dandruff remains obscure, the importance of *Malassezia* as a key contributor to the condition is widely recognized and has been comprehensively reviewed by Shuster [18]. The taxonomy of *Malassezia* has undergone extensive review since the initial identification of *Pityrosporum* yeasts by Malassez [8]. The application of phylogenetic techniques to the genera added further detail through the reclassification of the genus in the 1990s [19] to the current recognition of at least 14 distinct species of *Malassezia* [20–22]. The detail afforded by these taxonomic changes has in turn led to the recognition that *Malassezia globosa* and *Malassezia restricta* are the dominant species present on the human scalp [17, 23] and that these organisms may be substantially localized to the follicular infundibulum [24]. This is an intriguing observation, suggesting that the efficacy of particulate actives such as zinc pyrithione may, in part, be due to their effective delivery to this region [25, 26].

However, despite these advances in taxonomy, the precise mechanism by which *Malassezia* contributes to the characteristic inflammation, hyperproliferation and flaking response seen in people with dandruff, and the reasons why this response is confined to susceptible individuals have remained obscure despite several hypotheses having been presented in the literature, three of which are discussed in more detail below.

The potential causal link between the production of extracellular phospholipases by *Malassezia*.spp. and the occurrence of human and animal dermatoses has been highlighted in several studies. The ability of *M. furfur* to produce phospholipase A2 has been demonstrated *in vitro* by Plotkin *et al.* [27]. This hypothesis suggests that *Malassezia* may catalyse the production of arachidonic acid as a consequence of phospholipase A2 secretion and this may indirectly elicit an inflammatory response via the eicosanoid pathway. Interestingly, canine studies have also suggested a link between *Malassezia*-derived phospholipase activity and the formation of inflammatory dermatoses [28–30], indicating that *M. pachydermatis* recovered from the lesional sites of dogs suffering from canine dermatitis displayed greater phospholipase activity than samples collected from non-lesional sites, which in turn, displayed greater activity than *M. pachydermatis* collected from healthy dogs.

The relevance of such *in vitro* and canine models to human dandruff can of course be questioned and attempts to perform similar enzyme activity studies on human subjects have been confounded by the difficulties presented by the *in vitro* culture of scalp relevant *Malassezia* [31]. However, although no conclusive *in vivo* evidence is currently available, it is intriguing that an analysis of the *M.globosa* genome conducted by Xu *et al.* [32] highlighted that the genome of the dandruff relevant strain *M.globosa* possessed an unusually high copy number of secretory phospholipases.

Perhaps the most widely discussed and extensively researched of these hypotheses is that relating the onset of dandruff to the degradation of sebum triglycerides by *Malassezia*-derived lipases and the resultant accumulation of proinflammatory unsaturated fatty acids [33–35]. The initial work on this hypothesis [36] demonstrated that model sebum previously exposed to *Malassezia* spp. was capable of rapidly inducing an irritant reaction when subsequently applied to guinea pig skin. Following the taxonomic reclassification described previously, later work served to add considerable detail to this observation including the identification of numerous putative lipase genes in the *M. globosa* genome [32, 37, 38] and an incomplete β-oxidation pathway that may limit the utilization of unsaturated fatty acids, thus potentially driving an increase in the proportion of irritant unsaturated fatty acids present in human sebum [35, 39].

However, although this work has done much to improve our appreciation of the specific pathways of lipid metabolism present in *M. globosa* and *M. restricta,* the exact contribution of these metabolic proclivities to dandruff has yet to be demonstrated *in vivo*.

Several crucial questions need to be answered if this hypothesis is to be confirmed as the cause of dandruff. The relative contribution of *Malassezial* lipases to the total cutaneous lipase activity of the scalp in dandruff is currently unknown. This question is particularly pertinent given the observations that *Propionibacterium acnes* is the primary microbial source of cutaneous free-fatty acids [40–42] and therefore presumably of cutaneous lipases, and that the *P. acnes* population declines in dandruff and SD [43–45].

Given the observed reduction in *P. acnes* numbers and the observed reduction in free fatty acid levels [14, 46] in dandruff, the possibility must be considered that lipase activity may in fact be reduced in dandruff rather than elevated, as would be suggested by the unsaturated fatty acid hypothesis. In addition to the uncertainty regarding the contribution of *Malassezia* to the overall pool of free fatty acids, additional questions have been raised by recent work [47] demonstrating that *M. globosa* is capable of utilizing unsaturated fatty acids and therefore may be less capable of elevating the *in vivo* concentrations of unsaturated fatty acids than previously suspected.

Although most attempts to explain how *Malassezia* induce the flaking response observed in dandruff have focussed on putative roles for microbially derived phospholipases and lipases, recent work by Gaitanis *et al.* [20, 48] has suggested a potential role for a range of microbially derived bioactive indoles including malassezin, pityriacitrin and indolo[3,2-b]carbazole (ICZ) produced, almost exclusively, by *Malassezia* derived from subjects displaying symptoms of seborrhoeic dermatitis. Of these materials, ICZ was highlighted as being of particular interest because of its ability to act as a ligand of the aryl hydrocarbon receptor (AhR), suggesting the potential to elicit an irritant response *in vivo*; however, subsequent work using the physiologically relevant strain *M. globosa* [47] has raised questions about the likelihood of human scalp *Malassezia* activity as a significant source of ICZ *in vivo*.

To summarize the above paragraphs, although there remains a substantial consensus that *Malassezia*, along with individual susceptibility, is a key predisposing factor in dandruff and despite the enormous advances in our ability to characterize the genomic and metabolic characteristics of scalp relevant strains, a satisfactory explanation for the mechanism by which *Malassezia.* spp. provokes the dandruff response in susceptible individuals remains elusive.

## Stratum corneum and dandruff

### Stratum corneum structure and function

The classical features of dandruff, scaling and flaking, manifest in the surface layers of skin, the SC. To fully understand how this condition develops in susceptible individuals, it is important to understand the structure and organization of this highly specialized tissue and how these properties change during dandruff.

The SC acts as a barrier against pathogenic invasion by microorganisms, toxic agents, oxidants and ultraviolet radiation [49]. However, the most fundamental requirement of the SC is to act as a barrier to water loss, the so-called epidermal permeability barrier (EPB). Ultimately, loss of this function impacts many aspects of SC integrity and functionality [49]. Although essentially impermeable to water, a small but vital flux ensures that the tissue maintains its hydration and flexibility and thus its integrity and health. An essential mechanism that helps maintain water balance within the SC is the natural moisturizing factor (NMF) [50]. The NMF, a collection of low-molecular-weight water-soluble components, provides humectancy through effectively absorbing atmospheric water.

The SC is a multilayered tissue composed of anucleated, flattened corneocytes surrounded by multiple lamellar sheets of lipids. The SC structure has been likened to a brick wall in which the non-continuous, essentially proteinaceous terminally differentiated corneocytes (the bricks) are embedded within the continuous lipidic matrix (the mortar) formed by specialized lipids [51]. These lipids are unique in terms of composition, organization and physical properties. Ceramides (50%), cholesterol (25%) and fatty acids (10–20%) [52–54] represent the main elements, while other lipids present at lower levels (e.g. cholesterol sulphate) appear to play an important role as well [55]. Differences in the precise composition of the intercellular lamellar lipids, corneocyte size and shape, corneodesmosome number and SC thickness provide the structural basis for variation in permeability and cohesiveness seen in skin at different body sites [56, 57]. The scalp appears to be intermediate in thickness between facial and arm and leg skin [58].

The integrity of the SC is achieved through large numbers of specialized intercellular proteins called corneodesmosomes that lock neighbouring corneocytes together both in the plane of the SC layer and between adjacent SC layers [59, 60]. In healthy skin, the desquamation process is elegantly controlled to maintain tissue integrity and SC thickness by the coordinated activity of several classes of hydrolytic enzymes that act on the corneodesmosome [61–63]. Ultimately, water activity and the pH of the SC control these desquamatory enzymes [64]. Corneodesmosomes are the primary cohesive force that must be degraded to facilitate desquamation. Hydration of the surface layers is critical to facilitate desquamation, the process of skin turnover, which is so deranged in dandruff. Incorrect corneodesmosomal hydrolysis is a characteristic of many pathological skin disorders, including dandruff [15, 65, 66].

Sebum may partition deep into the intercellular SC lipids [67]. Triglycerides and short-chain fatty acids, while often listed as SC lipids, are primarily sebaceous in origin, and these molecules are hypothesized to play no significant role in EPB function. Rather, their presence may disrupt intercellular lipid organization close to the surface to facilitate desquamation [67]. In dandruff, sebum may actually perturb the desquamation process and impair lipid organization [15].

### Mechanisms of SC damage and repair

**Figure 2:**
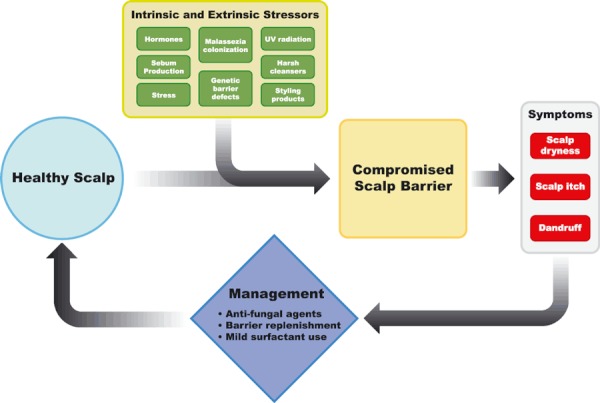
Model of barrier dysfunction in dandruff.

Intrinsic (disease) and extrinsic (temperature, low humidity and surfactants) factors can impair barrier function. There is a seasonal variation in structural barrier lipid levels [68] that may exacerbate winter xerosis. Psychological stress associated with increased circulatory stress hormones, which have been shown to delay EPB recovery, may further exacerbate the condition [69]. One or more of these factors can make the SC more prone to perturbation, potentially inducing dryness, irritation and itch (Fig. 2).

In normal homeostasis, minor acute EPB damage is ameliorated effectively. The loss of barrier function initiates a signalling cascade within the underlying epidermis that stimulates a repair response designed to normalize SC function [70]. The primary response is a temporary increase in the biosynthesis of major lipid species (i.e. ceramides, cholesterol and fatty acids) [71]. The so-called lamellar bodies responsible for the delivery of these lipids into the extracellular domains of the SC have also been shown to contain antimicrobial peptides vital to reducing pathogenic infections [72]. Hence, there is a coordinated restoration of both the epidermal permeability and the antimicrobial barriers following damage to the SC [73].

Severe or chronic EPB damage, however, can stimulate signalling pathways that evoke inflammatory events deeper in the skin, which may sustain inflammatory dermatoses [74, 75]. Perturbation of barrier function can lead to inappropriate epidermal proliferation (hyperproliferation) [76]. The decreased transit time, or turnover, of keratinocytes through the epidermis caused by hyperproliferation is associated with abnormal keratinization. This sequence of events creates an intrinsically inferior SC, continuing until the environmental insult is removed.

The defective SC barrier caused by severe or chronic damage is a critical element driving inflammation and should not simply be considered a secondary consequence of an underlying inflammatory condition (e.g. the ‘inside–outside’ model). In recent years, the critical importance of improving basic barrier function has become increasingly apparent to dermatologists treating inflammatory dermatoses, such as atopic dermatitis and psoriasis [77]. Indeed, Elias and co-workers have proposed that the abnormal water permeability in atopic dermatitis actually drives disease activity in what they call the ‘outside–inside–outside’ paradigm [78]. This hypothesis has gained prominence and increased acceptance with the elegant genetic studies conducted by McLean and others who have reported that the loss-of-function mutations in the unique epidermal protein filaggrin (a critical component of the SC) are associated with ichthyosis and atopic dermatitis [79, 80]. Thus, the importance of maintaining SC structure and function, epitomized ultimately by the quality and resilience of the EPB function, is clear.

### Perturbation of scalp stratum corneum in dandruff

In the context of this review, we have focused on the proposal that an inferior EPB function is pivotal to the initiation and/or exacerbation of dandruff by facilitating the ingress of *Malassezia* metabolites and other toxins of microbial origin. However, the ultimate manifestation of the underlying tissue damage as a poorly desquamating, flaky SC is likely to reflect many perturbations of the epidermis and SC of which an inferior EPB is just one consequence. In addition to studies that indicate a decreased epidermal transit time [44], the dandruff scalp SC shows striking features consistent with hyperproliferation: parakeratotic nuclear retention, irregular corneocyte structure, intracellular lipid droplets and loss of the organized lamellar lipid structure [15]. Specifically in relation to the EPB, the levels of competent barrier lipids have been shown to be dramatically depleted in dandruff [14]. The mass of poorly organized intercorneocyte lipids observed by electron microscopy in dandruff flakes is probably sebaceous in origin, providing little permeability barrier benefit. This observation is consistent with the poor barrier quality characterized by the dramatically increased TEWL measured in the SC of dandruff sufferers [81]. A complete loss or reduced levels of corneodesmosomes have also been reported in the dandruff scalp SC [15], although intuitively this observation is inconsistent with the increased physical cohesion observed between corneocytes. It is reasoned that the altered corneocyte morphology, disorganized intercellular lipids and increased sebaceous lipids may dramatically increase intercorneocyte cohesion.

The preliminary characterization of the dandruff scalp SC has indicated some similarities with winter- or surfactant-induced dry skin (xerosis) [60]. However, in comparison with dandruff, the depletion of intercellular lipids in dry skin is modest and other features such as parakeratosis are rarely observed in winter xerosis, observations that imply the dandruff scalp SC phenotype represents a more severe perturbation of epidermal function, keratinization, and overall barrier integrity than classical dry skin seen elsewhere on the body. Given these observations on the dandruff condition, it is likely that other elements of the holistic SC barrier are perturbed, for example the antimicrobial and antioxidant barrier.

Consistent with the hypothesis of SC disruption, several observations about dandruff susceptibility are consistent with poor EPB function. First, only individuals prone to dandruff respond to the topical application of oleic acid (a known skin penetration enhancer and irritant) [33], and secondly, dandruff sufferers are more responsive (i.e. report a higher perception of itch) to histamine topically applied to the scalp [14, 82]. Individuals with healthy scalps only respond to topically applied histamine following ‘active’ delivery of the pruritogen into the scalp with iontophoresis, whereas dandruff sufferers report itch before the iontophoretic current is applied.

Similarly, it has recently been reported that elevated levels of histamine are present in dandruff scalp SC compared with healthy scalp SC [83]. Although dandruff is often associated with itch and histamine is a classical mediator of itch, the direct involvement of histamine in eliciting natural scalp itch remains unproven. Nevertheless, pruritus is a common feature of dandruff, and involuntary scratching provoked by severe itching can further exacerbate the intrinsic weakness of the barrier through a physical disruption of the SC.

Furthermore, several studies have reported the detection of elevated levels of pro-inflammatory cytokines (interleukin-1α [84] and tumour necrosis factor α [85]) in the dandruff scalp SC. These cytokines are readily measured in superficial tape strips of the SC and are consistent with chronic perturbation of barrier function eliciting inflammatory and immunological responses within the underlying epidermis that initiate, maintain or exacerbate the dandruff condition.

## Antidandruff therapies

The goal of cosmetic antidandruff treatment is to restore the scalp to a healthy condition. In the United States, the Food and Drug Administration (FDA) recommends agents that reduce *Malassezia* levels (e.g. antifungals) or facilitate the removal of flakes (e.g. keratolytic agents or combinations of anti-inflammatory and antiproliferative agents with antimicrobials). Similar strategies are employed throughout the world to reduce dandruff-associated flaking and pruritus.

### Measuring the severity of dandruff

In the clinical setting, dandruff is measured visually by a trained expert using a validated assessment protocol [46]. The scalp is examined by quadrant under standardized lighting conditions. Each quadrant is rated for dandruff severity (Table I) and analysed statistically. The antidandruff efficacy of test products is assessed by examining the reduction in visual dandruff. This score is then compared with a suitable control. The longevity of antidandruff efficacy can be examined by placing subjects on a non-antidandruff shampoo at the end of the study test phase and monitoring for the return of dandruff flakes (regression phase).

**Table I:** Definition of severity grades and scores for adherent scalp flaking

Severity description	Description	Severity grade score
O	Healthy scalp with no dryness or dandruff	0
A	Fine dryness on scalp surface	1
B	Small powdery flakes partially adhering to scalp	2
C	Moderately flaky scales loosely attached to scalp	3
D	Large pronounced crusty scaling adhering to scalp	4
E	Very large crusty scaling congealed into plates adhering to scalp	5

### Antifungal treatments

The spectrum of antifungal agents used in dandruff treatments differs between countries. Zinc pyrithione, selenium sulphide, sulphur and ketoconazole are used in the United States as over-the-counter (OTC) applications, while other regions of the world may also use alternative antifungals such as imidazoles (e.g. climbazole) or hydroxypyridones (e.g. piroctone olamine and ciclopirox) either alone or in combination with FDA-registered actives (Table II). When trying to compare the relative clinical efficacy of antidandruff treatments, it is important to take into account several critical variables such as inclusion level of the active ingredient, the intrinsic antifungal potency and the efficiency of deposition and retention onto scalp skin. In our opinion, to date, no definitive single, well-controlled study of the known antidandruff treatments has addressed these variables. Consequently, no conclusion on the relative efficacy of the commonly applied treatment options is available.

**Table II:** FDA antidandruff active agents

Agent	Approved use	Mechanism
Zinc pyrithione	Dandruff Seborrhoeic dermatitis	Antifungal Inhibition of fungal growth Depletes intracellular ATP levels [86] Disrupts metal homeostasis [87, 88]
Selenium sulphide	Dandruff Seborrhoeic dermatitis	Antifungal Inhibition of fungal growth Antiproliferative [89, 90]
Coal tar	Dandruff Seborrhoeic dermatitis	Reducing agent Antipruritic Antiproliferative [89]
Salicylic acid	Dandruff Seborrhoeic dermatitis	Keratolytic agent Reduces epithelial hyperproliferation Improves pruritus

Zinc pyrithione is unquestionably the most widely used of the available active agents, offering an effective combination of antimicrobial potency, high efficiency deposition from modern shampoo systems and a demonstrated ability to deliver active material into the hair follicle [25, 26], enhancing the ability of the active agent to target both scalp surface and infundibular *Malassezia* [24]. Although antifungal activity is a key driver of efficacy, it has long been recognized [18] that many of the known antidandruff agents may also have non-antifungal effects that may contribute to the overall clinical benefit. Examples of this are the cytostatic effects ascribed to selenium sulphide and the intrinsic anti-inflammatory properties of ketoconazole [89].

### Opportunities for new strategies to maintain scalp health

Although there is no doubt that treatments such as zinc pyrithione and climbazole are effective antifungals, they do not directly address the deleterious changes in SC biology as measured by increased TEWL and lipid levels observed in individuals with dandruff [14]. Although a normalization of scalp barrier lipids is reported to accompany zinc pyrithione treatment [46, 84, 91], this effect is believed to be an *indirect* change arising from the removal or reduction of the microbial irritant. Thus, the reduction in microbial challenge *subsequently and indirectly* leads to the recovery and normalization of cellular processes, including keratinocyte proliferation and lipid biosynthesis, which in turn allow the underlying epidermal layers to recover and gradually repair the SC barrier over a period of days or weeks. Given the indirect nature of antifungal-derived EPB repair, the opportunity exists to drive a more immediate and selective barrier replenishment strategy through the topical application of lipids to *directly* replenish the SC and deliver a more rapid restoration of barrier quality.

In addition, shampooing with harsh surfactants will undermine the efficacy of conventional cosmetic antidandruff treatments by repeatedly damaging the SC permeability barrier already compromised by the reduction in lipid content apparent on the dandruff scalp. Such damage will manifest itself as after-wash tightness, dryness, barrier damage, irritation and itch.

Harsh surfactants, with their ability to damage lipids and proteins, are closely associated with loss of SC barrier lipids, water-soluble NMF and a reduction in the enzyme activity fundamental to the normal functioning of the SC [92, 93]. Collectively, these changes in SC composition impact the overall barrier quality, impair desquamation and promote flaking.

**Figure 3:**
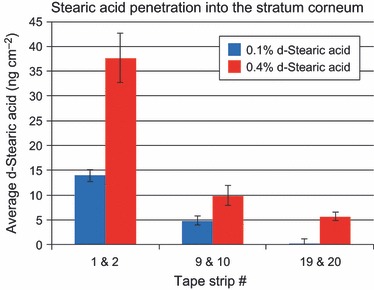
Stearic acid penetration into stratum corneum following single application of shampoo containing zinc pyrithione (1%) and deuterated stearic acid.

However, over the past 20 years, the development of cleansing products based on increasingly mild surfactants has led to a significant reduction in the SC damage that occurs during cleansing. Increasingly, mild liquid cleansing technology uses a combination of anionic and amphoteric surfactants to reduce protein damage and the intrinsic skin irritation potential of anionic surfactants [94]. Nevertheless, this combination can still result in skin dryness, probably as a result of the interaction of surfactants with skin lipids. The most recent advances in skin cleansing technology have demonstrated that pH-neutral synthetic detergent-based bars and body washes containing sodium alkyl isethionate are particularly gentle to skin [92, 93, 95]. Washing skin with a liquid cleanser base (in the absence of any moisturizing ingredients) can reduce the level of fatty acids and cholesterol in the skin even after a single wash. Transmission electron micrographs of human skin washed *ex vivo* under exaggerated conditions have shown that non-ionic surfactant-based cleansers alter the lipid region to a greater extent than do mild cleansing bars with sodium cocoyl isethionate as the surfactant [96]. In such mild systems, the addition of saturated long-chain fatty acids, such as stearic and palmitic acid, lowers the tendency of the surfactant to damage lipid membranes. This is perhaps due to their ability to act as a buffer against lipid extraction by surfactant micelles and to deposit and replenish some of the fatty acids lost during cleansing [93]. It has been reported that stearic acid can, with effective formulation and skin deposition, become incorporated into all layers of the SC [97]. Replacing intrinsic fatty acids inevitably lost from the skin surface during the washing process can help improve barrier function as measured by TEWL and other physiologically relevant assessments. Although the data published to date have been generated on face and body skin, we believe that the findings are equally relevant in the case of scalp cleansing with shampoo formulations. We have recently demonstrated that such technical approaches also replenish fatty acids within the scalp when delivered from shampoos. Recent studies in our laboratory using deuterated stearic acid have demonstrated the ability to deliver this fatty acid into the SC from a fully formulated antidandruff shampoo (Fig. 3). Delivery of stearic acid to the SC parallels the improvements in self-assessment of skin health performed by study participants. Such an approach can be further complemented by the use of polar moisturizing oils, such as triglyceride oils, that reduce surfactant binding to proteins and in turn make the cleanser milder towards skin [95]. These properties further enable the SC to maintain intrinsic moisture and enzymic functionality, both vital to improving the dandruff condition. We believe that treatment of dandruff with multi-functional shampoos that both directly promote the integrity of the SC and provide effective antifungal activity may thus be beneficial.

## Conclusions

In this review, we discussed the key factors in the development of dandruff. Growth of *Malassezia*, likely nourished by sebum, has long been associated with dandruff scalps, but the precise mechanisms underpinning the relationship between *Malassezia* and dandruff remain unclear. Despite this uncertainty, one of the more reliably observed features of the condition is that dandruff develops only in susceptible individuals, suggesting that additional factors may make the scalp epidermis vulnerable to damage from the growth of *Malassezia* and additional stressors. Furthermore, the weaker epidermal permeability barrier may be more susceptible to surfactant damage occurring through frequent shampooing.

Defects in the SC permeability barrier may underlie this individual susceptibility. Internal and external stressors can disrupt the SC structure, reducing the levels of NMF and all classes of free lipids. We propose that this weakened SC structure initiates or exacerbates the atypical desquamation and pruritus characteristic of dandruff. Current antidandruff formulations indirectly treat the underlying SC dysfunction by reducing levels of *Malassezia* colonization and consequent production of metabolites. Additionally, delivery of saturated fatty acids to the SC from mild surfactant formulations may directly replenish and strengthen the epidermal barrier. We propose that advanced shampoos that combine mild surfactants, direct barrier lipid replenishment and antimicrobial activity may optimize scalp health in individuals with dandruff more comprehensively and effectively than either approach alone.
